# Comparing the Impact of Upper Body Control and Core Muscle Stabilization Training on Landing Biomechanics in Individuals with Functional Ankle Instability: A Randomized Controlled Trial

**DOI:** 10.3390/healthcare12010070

**Published:** 2023-12-28

**Authors:** Daekook M. Nekar, Dong-Yeop Lee, Ji-Heon Hong, Jin-Seop Kim, Seong-Gil Kim, Yeon-Gyo Nam, Jae-Ho Yu

**Affiliations:** Department of Physical Therapy, Sun Moon University, Asan 31460, Republic of Korea; daekooknek@gmail.com (D.M.N.); sgkim4129@sunmoon.ac.kr (S.-G.K.);

**Keywords:** functional ankle instability, upper body control, core stabilization training, landing biomechanics

## Abstract

Functional ankle instability (FAI), which is characterized by recurrent ankle sprains and perceived joint instability, arises from various factors contributing to compromised biomechanical control during activities, particularly those involving landing tasks. While current research predominantly addresses lower-extremity and core stabilization interventions for FAI, the contribution of upper body control to landing biomechanics in this population remains insufficiently explored. In this study, 42 participants (19 males, 23 females) with FAI were randomly assigned to either the upper-body control training group (UBCTG) or the core muscle stabilization training group (CMSTG). The groups underwent six-week interventions, with the UBCTG receiving a dynamic core exercise program including upper body control and the CMSTG receiving static core muscle training. Pre- and post-intervention assessments encompassed electromyography of the gastrocnemius, tibialis anterior, and peroneus longus, motion analysis of the lower extremities, and ground reaction force (GRF) readings during a single-leg-jump task. Additionally, dynamic balance was assessed using the Y balance test and self-reported measurements of ankle instability were performed. The results showed similar increases in muscle activation, joint movement, and self-reported ankle instability scores within both groups. However, significant between-group differences were observed in terms of knee flexion angle, dynamic balance, and ankle instability scores, favoring the UBCTG. Although the peak vertical GRF significantly decreased and the time to peak vertical GRF increased in both groups, more changes were noted in the UBCTG. Our results demonstrated that dynamic core exercises with additional upper body control training enhance landing biomechanics, dynamic balance, and stability in individuals with FAI. Consequently, we recommend incorporating shoulder girdle exercises, proprioceptive drills, and balance exercises into dynamic core training.

## 1. Introduction

Functional ankle instability (FAI) is a prevalent musculoskeletal issue characterized by recurring ankle sprains and a persistent perception of joint instability, which affects individuals across diverse demographics and athletic backgrounds [1,2]. The multifactorial etiology of FAI involves neuromuscular deficits, ligamentous laxity, and altered proprioception, leading to compromised biomechanics during weight-bearing activities, particularly dynamic landings [3,4].

Ankle sprains, a primary contributor to FAI, often occur during activities requiring precise control over landing biomechanics, such as cutting maneuvers, jumps, and sudden changes in direction [5,6]. Traditional interventions primarily focus on lower-extremity factors, emphasizing strengthening exercises, proprioceptive training, and balance exercises [7]. However, recognizing the pivotal role of pelvic and trunk stability in facilitating bodily movements highlights the significance of the core muscles, which initiate activity-preceding movements in the lower and upper extremities [8,9,10]. Kibler et al. [9] emphasized that core stabilization helps the production of force conform to a proximal-to-distal pattern, leading to interactive moments and safe movement of the distal joints. Consequently, researchers have extended their investigations to the trunk and core muscles.

Evidence suggests that engaging in core stability exercises can effectively reduce ground reaction force (GRF). Arajo et al. [11] demonstrated that a six-week core stability training program resulted in a decrease in vertical GRF (vGRF), enhancing landing kinetics. Furthermore, Fatahi et al. [12] explored the effects of an eight-week core stability training program on kinetics during single-leg drop landings in healthy individuals, revealing a significant reduction in both maximum vGRF and loading rate. This indicates that sustained engagement in core stability exercises can positively impact biomechanics, potentially reducing the risk of lower-extremity injuries [12]. However, research on individuals with FAI has primarily focused on the effects of core stability programs on specific biomechanical outcomes. Existing studies have predominantly featured training programs incorporating a mix of exercises [13,14], either targeting specific core muscle groups [15] or solely focusing on changes in GRF during landing tasks with static core muscle stability exercise [16]. 

Given the importance of postural control for individuals with FAI, exploring the potential impact of dynamic exercises involving upper body control is of great interest. To date, the literature lacks insights into the contribution of the upper body to postural control, encompassing proprioception, balance, and coordination. Effective upper body control is recognized as being critical for optimizing dynamic balance and load distribution during weight-bearing activities [17,18]. Therefore, understanding the nuanced interactions between upper body control and landing biomechanics may provide valuable insights into the holistic management of FAI. Furthermore, it is imperative to move beyond solely examining the biomechanical aspect of exercise outcomes toward considering functional impacts, especially since our targeted population comprises individuals with FAI. Notably, there is currently no study that incorporates a functional assessment following the implementation of a core/upper body exercise program.

The present study endeavors to address this research gap by investigating the impact of a dynamic exercise program integrating training for upper body control on individuals with FAI. By leveraging evidence-based strategies targeting both lower and upper body dynamics, we aim to elucidate the potential contribution of the upper body to landing biomechanics and functional outcomes in individuals with FAI. By undertaking this comprehensive investigation, we aim to contribute valuable data to the existing body of knowledge, with potential implications for refining rehabilitation protocols and injury prevention strategies that are tailored to the intricate demands of FAI. Ultimately, our study seeks to advance the understanding of the role of upper body control in the biomechanics of landing tasks, providing a foundation for more effective interventions in the management of FAI. Our hypothesis postulates that the integration of dynamic exercises, specifically those targeting upper body control, alongside core stabilization exercises will lead to a notable improvement in landing biomechanics, dynamic balance, and overall functional outcomes among individuals with FAI.

## 2. Materials and Methods

### 2.1. Study Design

This study employed a randomized controlled trial design with a parallel-group allocation ratio of 1:1. To allocate participants to either the upper body control training group (UBCTG) or the core muscle stabilization training group (CMSTG), randomization was achieved through computer-generated random numbers. The random allocation software (version 1.0.0) developed by Dr. Mahmood Saghaei [19] was employed for this purpose. This freely available software allows for both simple and blocked random allocation. In our study, we opted for a simple randomization method, with equal-sized random numeric generation to mitigate the risk of selection bias and ensure a reliable randomization process. Data collection for all specified outcome measures was conducted twice, once before and once after the intervention. To fortify the objectivity of the assessments, all measurement procedures were carried out by assessors who were intentionally blinded to the intervention status of the participants. The study adhered to the Consolidated Standards of Reporting Trials (CONSORT) guidelines [20].

### 2.2. Ethical Considerations

The study adhered to the principles outlined in the Declaration of Helsinki [21]. Informed consent was obtained from all participants, and ethical approval was obtained from the institutional review board of Sun Moon University (approval ID: SM-202104-035-1).

### 2.3. Participants

A total of 42 participants (19 males and 23 females; age range: 18–40 years) with diagnosed functional ankle instability (FAI) were recruited for this randomized quasi-controlled trial. The diagnostic process involved a comprehensive examination, commencing with a detailed inquiry into the patient’s history of ankle sprains, instability, and associated symptoms. Subsequently, to provide a subjective assessment, participants completed the Cumberland ankle instability tool (CAIT) assessment, which has a test-retest reliability of ICC2,1 = 0.91, a sensibility of 82.9%, and a specificity of 74.7% [22]. We specifically used the Korean version of CAIT (CAIT-K), which demonstrates strong test-retest reliability (ICC2,1: 0.95, SEM = 1.83 for right limbs, 0.96, SEM = 1.50 for left limbs) and Cronbach’s alpha coefficients of 0.92 and 0.90 for the right and left limbs, respectively, confirming its validity and reliability [23]. Supplementary to this testing, specific functional tests, including the anterior drawer test (with a sensitivity of 86% and a specificity of 74%) [24] and the talar tilt test (with a sensitivity of 50 and a specificity of 88%) [5], were administered. This multifaceted approach ensured a thorough evaluation of each patient’s condition, incorporating both subjective self-reporting and objective clinical assessments.

The sample size was determined using G*Power software (version 3.1.9.6) under the following configurations: *t*-tests, an effect size corresponding to Cohen’s d for a large effect (0.80), a significance level (α) of 0.05, and a desired statistical power of 0.80. The determined sample size was 42 participants, evenly distributed, with 21 individuals per group. Participants were selected based on clinical assessments and a reported history of recurrent ankle sprains. Exclusion criteria included previous surgery on the ankle, neurological disorders, and other musculoskeletal conditions affecting lower-extremity function. Participants were randomly assigned to either the UBCT group or the CMST group. Informed consent was obtained from all participants, following the institutional review board guidelines.

### 2.4. Intervention

#### 2.4.1. Upper Body Control Training Group (UBCTG)

Participants in the UBCTG underwent a 6-week intervention consisting of dynamic core muscle training along with targeted exercises to enhance upper body control. The upper body control exercises included resistance training for the shoulder girdle, proprioceptive drills, and balance exercises. The core training component incorporated movements and resistance training tailored to enhance stability and function in the upper trunk and shoulder girdle. The exercises were selected based on previous studies and were purposely adapted for the specific context of this investigation [25,26,27]. The intervention was performed three times a week, with each session lasting 30 min. 

#### 2.4.2. Core Muscle Stabilization Training (CMST)

Participants in the CMST performed 6 exercises focusing on core muscle stabilization, mainly comprising static exercises contributing to overall core stability. The upper body was not the primary focus of the intervention for this group. The exercises were chosen from those in the study by Pirmohammadi et al. [16], in which the authors used the McGill core muscle endurance tests to assess core muscle endurance and the control effectiveness of core stability training (Table 1). The differences between the UBCT and CMST methods hinge on the nature of the exercises, specifically, whether they were dynamic or static and regarding the engagement of the upper limbs. Visual representations of each exercise are provided in Supplementary File S1. The intervention was performed three times a week, with each session lasting 30 min.

### 2.5. Outcome Measure

#### 2.5.1. Biomechanical Assessments

**Figure 1:**
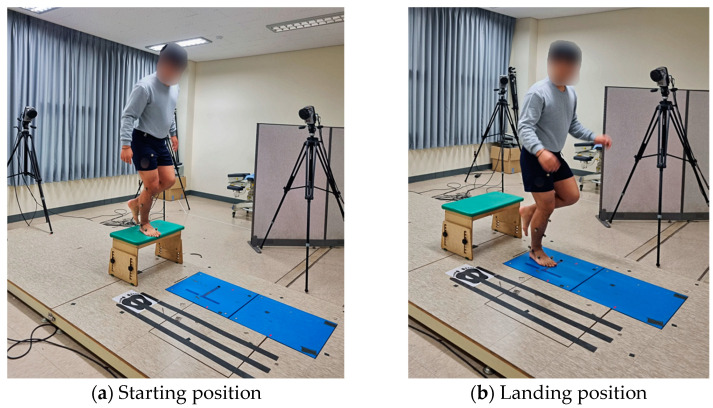
Motion capture during the single-leg-jump task.

The biomechanical assessments encompassed electromyography (sEMG) and the motion analysis of joint movements and ground reaction force, with all assessments conducted during a single-leg-jump activity. To closely replicate the conditions under which ankle sprains commonly occur, such as during jumping or running, a horizontal jump procedure was selected. Participants were instructed to stand on their designated leg, fully extending the knee, on a platform elevated to a height of 30 cm. They were then required to execute a forward jump, landing on the same leg on a ground force plate. Following landing, the participants were instructed to maintain an upright and forward-facing trunk position for a minimum of 5 s. The jump task was performed barefoot, and no specific guidelines were provided regarding the placement of the upper extremities. In instances where the alternate leg contacted the force plate or if the participant was unable to maintain stability, the measurement was deemed invalid and was recorded as an unsuccessful jump. To familiarize participants with the task and ensure their comfort, practice sessions were administered. A rest period was also provided before the commencement of the actual data collection, to optimize the participants’ readiness and overall performance. An illustration of the single-leg-jump test is presented in Figure 1.

The surface EMG (sEMG) of the gastrocnemius lateralis (GCM), tibialis anterior (TA), and peroneus longus (PL) of the injured leg were recorded. The selection of the GCM, TA, and PL muscles for sEMG recording in the injured leg is grounded in their pivotal roles in ankle stability, control, and movement. Previous research has highlighted instances of weakened activation, dysfunction, or delayed reaction time in the PL (a key contributor to ankle eversion and plantarflexion), the TA (an essential dorsiflexor), and the GCM (a significant plantar flexor) in relation to ankle sprains and instability [28,29,30,31,32]. 

To establish a baseline for normalization before recording the sEMG data during the jump task, 2 sets of 5-second recordings were conducted for each muscle, capturing the maximum voluntary contractions (MVC). Participants engaged in these contractions against manual resistance, utilizing the Lafayette hand-held dynamometer. Details of the measurement position have been published elsewhere [33]. The sEMG was then recorded with dual self-adhesive Ag/AgCl gel surface electrodes (Noraxon Dual EMG Electrode, diameter 10 mm, inter-electrode distance 17.5 mm) and the data were acquired using the AURION ZeroWire device (Milan, Italy) at a sampling rate of 1000 Hz. Subsequent analysis of the raw EMG data was conducted using MyoRESEARCH software (XP Master, version 1.07.1, Noraxon, Scottsdale, AZ, USA). The subsequent analysis of the raw EMG data utilized MyoRESEARCH software (XP Master, version 1.07.1, Noraxon, Scottsdale, AZ, USA). The signal underwent bandpass filtering between 20 and 450 Hz, while further processing involved root mean square (RMS) calculations with a window of 10 ms, including full-wave rectification and smoothing. To minimize skin resistance and crosstalk, skin preparation (shaving and cleaning) and electrode placement followed SENIAM recommendations, aligning the electrodes with the muscle fiber orientation on the muscle belly.

The kinematic data were assessed using motion analysis, employing a three-dimensional motion capture system (Qualisys system-Qualisys Medical AB 41113, Gothenburg, Sweden, Qualisys Oqus 300). A total of 15 reflective markers were strategically placed on the trunk and upper extremities, following the upper extremity marker placement protocol. The utilization of a 6-camera motion analysis system allowed for the capture of kinematic data, contributing to the identification of potential compensatory movements that might be associated with functional ankle instability.

The assessment of ground reaction forces during landing tasks utilized the Kistler 9286AA force platform (Kistler, Winterthur, Switzerland), employing a free measurement approach and not using predefined tests included in the software. The force platform is instrumental in capturing and quantifying the intricate dynamics of forces acting on the lower extremities during landings. The recorded data from the Kistler 9286AA force platform measuring vGRF, along with those collected by Qualisys Oqus 300 cameras tracking the positions of retroreflective markers, were exported as C3D files and then analyzed using the Visual 3D (C-Motion, Germantown, MD, USA).

#### 2.5.2. Functional Assessments

Dynamic balance was assessed using the Y balance test, a widely used clinical tool employed to assess the participants’ dynamic balance and lower extremity neuromuscular control (inter-rater reliability ICC = 0.85 to 0.93 and test-retest reliability ICC 0.80 to 0.85) [34]. The participants were briefly briefed on the test, with an emphasis on the importance of reaching as far as possible while maintaining balance. Subsequently, a researcher demonstrated the test by performing 2 practice trials, illustrating the proper technique and balance. Participants were then instructed to stand on the affected leg at the center of the grid, with the other foot raised and poised to reach in different directions. Three trials for each reach direction were performed and the mean value was used. Commonly, 3 trials were conducted in each direction for each leg. Normalization of the distance reached was achieved by dividing the measured distance by that participant’s leg length.

The CAIT-K approach was utilized to assess the severity of self-reported ankle instability. The questionnaire was cross-culturally translated and adapted into Korean from the original version according to the accepted guidelines [23]. The CAIT-K consists of 9 statements that are chosen to ascertain a 30-point score, with a lower score indicating severe ankle instability. This self-report questionnaire has a test-retest reliability of ICC2,1 = 0.91, sensibility of 82.9%, and specificity of 74.7% [22]. Participants were asked to rate both their legs by choosing the statement in each item that most accurately characterized the condition of the respective ankle joints. 

### 2.6. Statistical Analysis:

The SPSS statistics software, version 29.0, for Windows (Armonk, NY, USA: IBM Corporation) was used for statistical analysis. Descriptive statistics was used to summarize the participant characteristics. The Shapiro–Wilk test was used to analyze the normal distribution of data. For normally distributed data, the between-group comparisons of variables were conducted using independent *t*-tests. Changes over time within each group were assessed using paired sample *t*-tests. For non-normally distributed data, the Wilcoxon signed-rank test was used for the within-group comparison, and the Mann–Whitney U test was employed for the between-group comparison. The level of significance was set at *p*  <  0.05.

## 3. Results

### 3.1. Demographic Information

The demographic details of the participants are outlined in Table 2. No significant differences were observed between the groups concerning age, weight, height, body mass index, and CAIT-K score. It is noteworthy that all participants had experienced a unilateral ankle sprain, with the right limb being predominantly affected. Importantly, all participants successfully completed the training program, and there were no dropouts.

### 3.2. Biomechanical Outcomes

#### 3.2.1. Muscle Activation

**Figure 2:**
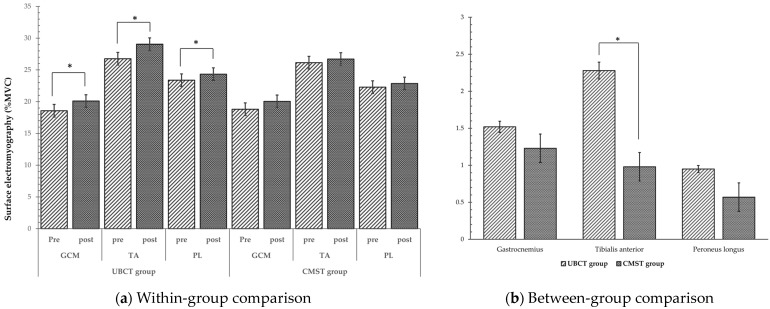
Comparison of muscle activation results within and between groups, * *p* < 0.05.

The results of the sEMG amplitude of all the tested muscles are presented in Table 3. The paired sample *t*-test demonstrated an increase in GCM, TA, and PL in the UBCT group, with a statistically significant difference (*p* < 0.05). Conversely, increases in the mean were observed between the pre- and post-test sEMG results of all muscles in the CMST group but were without any significant difference (*p* > 0.05). Regarding the between-group comparison, a statistically significant difference was observed in the TA with a *p*-value of 0.017. However, no significant difference was observed between the groups in the GCM and PL muscles (*p* = 0.766, *p* = 0.394, respectively). Figure 2 visually compares muscle activation both within and between groups.

#### 3.2.2. Motion Analysis

**Figure 3:**
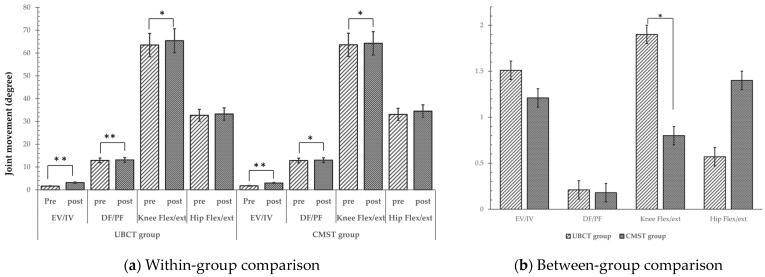
Comparison of joint movement results within and between groups, * *p* < 0.05, ** *p* < 0.001.

In terms of joint movements, the UBCT group showed an increase in joint angle mean in all the measured joints. We observed a statistically significant increase in ankle inversion/eversion, dorsiflexion/plantar flexion (*p* < 0.001), and knee flexion/extension (*p* < 0.05), but found no significant difference in the hip flexion/extension joint angles (*p* > 0.05). The CMST group, on the other hand, showed a significant increase in ankle eversion/inversion (*p* < 0.001), ankle dorsiflexion/plantar flexion, and knee flexion/extension (*p* < 0.05). Despite an increase in mean value, no significant difference was observed in hip flexion/extension (*p* > 0.05). The between-group comparison showed a significant difference in knee flexion/extension, with a higher increase in the UBCT group (*p* = 0.033) (Table 4). Figure 3 provides a visual comparison of joint movement, illustrating both within-group and between-group comparisons.

#### 3.2.3. Ground Reaction Force

**Figure 4:**
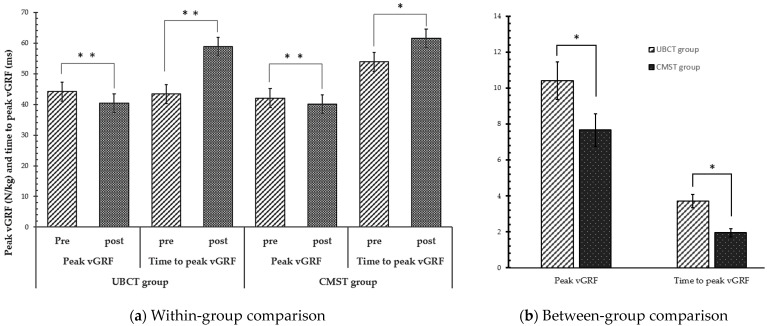
Changes in vertical ground reaction force and the time to peak vertical ground reaction force, * *p* < 0.05, ** *p* < 0.001.

The peak vGRF and the time to peak vGRF during the landing tasks performed by both groups are presented in Table 5 and Figure 4. The UBCT group showed a significant decrease in peak vGRF and an increase in the time to peak vGRF between the pre- and post-test values (*p* < 0.001). Similarly, the CMST group demonstrated a significant decrease in peak vGRF (*p* < 0.001) and an increase in the time to peak vGRF (*p* < 0.05). In terms of a comparison between the groups, significant differences were observed in both the peak vGRF (*p* = 0.045) and the time to peak vGRF (*p* = 0.020). 

### 3.3. Functional Outcomes

**Figure 5:**
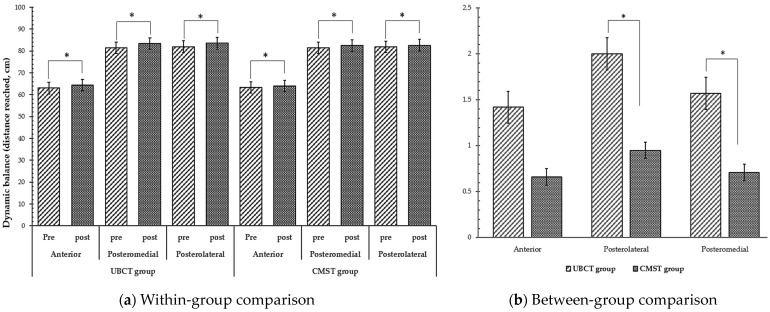
Comparison of muscle activation results within and between groups, * *p* < 0.05.

Regarding the functional outcomes, significant changes have been observed in both groups in the dynamic balance test (Table 6) (Figure 5). Both the UBCT and the TLBT groups showed a statistically significant increase in all directions (anterior, posterolateral, and posteromedial) where *p* < 0.05. During the between-groups comparison, we observed significant differences in posterolateral and posteromedial results, with *p* = 0.034 and *p* = 0.027, respectively.

The results of the ankle instability severity test are presented in Table 5. Both groups showed a significant increase between the pre- and post-test values (*p* < 0.05), while the between-group comparison showed a significant difference between the groups (*p* = 0.044).

## 4. Discussion

The purpose of this study was to explore the contributions of upper body control to the biomechanics of landing tasks in individuals with FAI. This study specifically investigates the impact of a dynamic upper body control training program on landing biomechanics and functional outcomes in individuals diagnosed with FAI. The findings of the current study illuminate the effects of a dynamic upper body control training program on muscle activation, joint movements, and ground reaction forces during landing tasks, dynamic balance, and the severity of FAI. 

### 4.1. Biomechanical Properties

#### 4.1.1. Muscle Activation

In the present study, the sEMG amplitude increased in GCM, TA, and PL within the UBCT group, with statistically significant improvements. The observed changes suggest the positive impact of upper body control training, including dynamic core stabilization exercises, on lower limb muscle activation. Notably, these findings deviate from previous research affirming that upper-body exercise may not influence muscle activation, jumping performance, and post-activation performance enhancement in the lower body [35]. The results of this previous study are understandable when considering that without explicitly targeting lower limb muscles in the training program, achieving statistically significant activation in these muscles could be challenging. Nonetheless, during the execution of upper body control training in our study, the participants required a stable and firm base of support while engaging their core muscles, as well as the upper extremities. This training inherently involved the activation of certain lower-limb muscles, contributing to the observed trend in muscle activation. It is essential to mention that during postural control, the ankle strategy predominantly involves the ankle muscles, including the TA or GCM [36]. Therefore, we can assert that engaging the upper body can influence the activation of lower-body muscles, depending on the position in which the exercises are performed.

In contrast, the CMST group exhibited changes without showing a statistically significant improvement in lower-body muscle activation. The observed changes suggest the limited positive impact of the static core stabilization exercises on lower-limb muscle activation. These results do not align with those of a previous study conducted on healthy athletes, in which similar core exercises resulted in increased hip and knee muscle activation [37]. This disparity may be attributed to the longer intervention time in the previous study. Compared to the UBCT exercises, the CMST exercises were performed in supine or prone positions that inherently do not involve a large number of lower body muscles. This finding underscores the distinct responses elicited by the two interventions, emphasizing the importance of a targeted emphasis on lower-body training and lower-limb muscle activation.

#### 4.1.2. Joint Movements

Regarding joint movement, the significant increases in joint angles in ankle inversion/eversion, dorsiflexion/plantar flexion, and knee flexion/extension in the UBCT group demonstrated a comprehensive effect on lower extremity kinematics. Notably, these changes were more pronounced in ankle and knee movements compared to hip flexion/extension. This result aligns with the existing literature, which reported a decrease in ankle dorsiflexion and knee movement in individuals with FAI, with an increase in hip flexion to compensate for and reduce the shock received during contact and the risk of injury [38,39,40]. The results of the present study demonstrate that upper body control reduces the need for compensatory movement in the hip joint and increases ankle dorsiflexion and knee flexion. This may be explained by the fact that incorporating dynamic exercises that involve not only core stability but also upper limb movement provides stability and increases coordination between movements. In their study, Sheikhi et al. [41] suggest that altered trunk and lower-body movements promote more coordination, indicating improved dynamic stability during landing tasks. The authors suggest that these coordination changes may contribute to improved stability during landing.

The CMST group demonstrated comparable changes in ankle and knee movements, with a notable distinction observed in ankle inversion/eversion. This outcome stems from the specific nature of the intervention methods, emphasizing the impact of lower body training on the ankle in both the sagittal and frontal planes. The intervention’s targeted approach to lower limb exercises likely contributed to the observed improvements in ankle kinematics. Interestingly, there was a lesser degree of change in knee flexion/extension compared with the UBCT group. This suggests that participants in the CMST group may have had relatively less proximal stability, resulting in a more moderate increase in knee joint movement. The specificity of the lower-body-focused training might also have influenced the participants’ stability profile, highlighting the intricate relationship between proximal and distal joint movements in response to targeted interventions.

#### 4.1.3. Ground Reaction Force

Both groups demonstrated significant decreases in peak vGRF and an increase in the time to peak vGRF, which is indicative of enhanced force distribution during landing tasks. These findings align with the concept that improved muscle activation and joint movements contribute to more effective force absorption. For example, Fong et al. [42] and Hoch et al. [39] found a strong relationship between dorsiflexion ROM and peak vGRF during landing and asserted that individuals with a greater ROM in dorsiflexion tend to land with a less upright posture, employing increased sagittal-plane displacement. This adjustment enables the body to absorb and dissipate forces more efficiently during landing.

The findings from prior studies using core stability exercises align with the results obtained in this study, indicating that core stabilization exercises offer proximal stability for enhanced distal mobility [16]. However, the disparities between groups in terms of both peak vGRF and the time it takes to reach this peak underscore the influence of UBCT on the generation and absorption of GRF during landings. This implies the potential superiority of UBCT over the static core stability exercise. In a previous study, the authors had identified that core stability training could diminish the forces applied to the joints of the lower extremities in single-leg drop landings [43]. Therefore, improved upper body control, including dynamic exercises adding to core stabilization, may lead to more effective load distribution across the lower extremities during landing. This can result in a smoother transfer of forces, potentially reducing the abruptness of force application and, consequently, decreasing peak vGRF, as observed in the present study. This mechanism aids in maintaining the center of gravity at the base of support and effectively absorbing the forces generated in the distal regions. 

Another mechanical consideration that may contribute to these effects could be optimized joint mechanics. In other words, the upper-body-control exercises involve coordinated movements that engage both the upper and lower extremities. Enhanced coordination and proprioception can contribute to optimized joint mechanics, ensuring a more controlled and gradual absorption of forces during landing. This, in turn, may lead to a reduction in peak vGRF.

It is important to mention that the body constitutes a connected kinetic chain in which improvements in upper body control may positively influence the entire kinetic chain, creating a cascading effect on lower-limb biomechanics [44]. An extended time to reach the peak vGRF may be associated with a more gradual loading of the lower extremities. Improved control of the upper body can contribute to anticipatory muscle activation, including muscles in the lower extremities. This explanation aligns with recent evidence suggesting that the dynamic control of ankle stability is achieved through the feed-forward mechanisms of the central nervous system, as opposed to relying solely on feedback from peripheral reflexes [45]. Although Gage [15] affirmed that improved landing mechanics in individuals with FAI after core stability exercise programs result from an improved feedforward mechanism, the addition of dynamic upper body movement appears to introduce even more robust feed-forward feedback. As a result, this contributes to the establishment of a more balanced feed-forward feedback loop, fostering a more stable foundation for landing. Consequently, there is a potential reduction in the need for sudden and forceful muscle contractions upon ground contact, allowing for an extended duration over which the impact forces are applied.

### 4.2. Functional Properties

In terms of functional outcomes, we observed notable improvements in dynamic balance across all directions within both groups following the six-week intervention. The enhanced dynamic balance was characterized by increased reach distances, a factor indicating a positive progression in the ability to maintain stability during challenging movements. Based on the explanations and the hypnotized mechanics that improved landing biomechanics, we can argue that improved proximal postural stability is the principal reason for this finding. However, we observed that when incorporating dynamic UBCT, there is a significant difference between the static core stabilization exercise in terms of posterolateral and posteromedial movements. This superiority can be explained by the fact that the UBCT involved diagonal movements that simultaneously activate muscles in different directions. Upper-limb diagonal movement is effective in improving muscle strength, muscular endurance, balance, and fine motor control [46,47]. Moreover, the observed improvements in muscle activation, joint movements, and ground reaction forces in the UBCT group may be attributed to the dynamic nature of the exercises, which likely engaged both lower and upper extremities in a coordinated manner. Enhanced proprioception, balance, and coordination resulting from UBCT may contribute to improved lower extremity biomechanics, providing a more holistic approach to addressing functional ankle instability.

### 4.3. Limitations

This study has some limitations. The six-week duration of the intervention may be considered relatively short. Longer-term interventions and follow-up assessments could provide insights into the sustainability of the observed improvements over an extended period. It is essential to acknowledge that we did not have control over the participants’ overall physical activity levels outside of the intervention. The inherent variability in physical activity among the participants may have influenced the observed outcomes, introducing a potential confounding factor. Additionally, the absence of a control group and the restriction to a specific age range (18–40 years) of patients diagnosed with FAI may limit the broader applicability of the results to diverse populations. 

### 4.4. Clinical Implications

The current study adds to the existing literature by specifically addressing the role of dynamic upper body control training in individuals with FAI, a facet that has been understudied in previous research. The study’s findings have direct implications for the development of rehabilitation protocols tailored to individuals with FAI. Integrating dynamic upper body control exercises alongside traditional ankle training may offer a more comprehensive and effective approach. Clinicians and rehabilitation specialists may consider incorporating dynamic upper body control exercises into existing exercise programs to enhance the overall biomechanics and functional outcomes of individuals with FAI.

### 4.5. Future Directions

Future research should explore the long-term effects of dynamic upper body control training, considering factors such as the retention of improvements and potential injury prevention benefits. Research with larger cohorts could further validate and strengthen the current findings. It is essential to explore the transferability of these findings to real-world scenarios; broadening the age range could enhance the generalizability of the findings to a more representative spectrum of individuals. Future studies should not rely on pre- and post-intervention assessments without introducing intermediate evaluations during the six-week intervention period. Intermediate assessments could offer a more detailed understanding of the progression of biomechanical changes throughout the intervention. Furthermore, including a control group would enhance the internal validity of the study by allowing for better comparison and interpretation of the intervention’s effects. Examining both the spine and pelvis would be intriguing, enabling a comprehensive analysis of the body’s adaptations to compensate for the asymmetry found in unilateral FAI. Finally, future research should consider a factorial approach to identify the most contributory exercises or the optimal combinations that would maximize their effectiveness.

## 5. Conclusions

In conclusion, this study sheds light on the significant contribution of upper body control to core stabilization training on muscle activation, joint movements, ground reaction, dynamic balance, and ankle instability in individuals with FAI. To provide a more explicit recommendation, we emphasize incorporating exercises targeting the shoulder girdle, proprioceptive drills, and balance exercises within a regimen of dynamic core stabilization exercises. By doing so, rehabilitation protocols can be tailored to address both upper and lower body biomechanics, enhancing the overall stability, joint movements, and dynamic balance of individuals with FAI. These results emphasize the pivotal role of upper body engagement in refining rehabilitation strategies for comprehensive FAI management. 

## Figures and Tables

**Table 1:** Intervention program.

UBCT Group	CMST Group
Resistance Band Pallof Press	Plank
Medicine Ball Slams	Side Plank
Rotational Medicine Ball Throws	Supine Bridge
Diagonal Lunge with Twist	Abdominal Crunch
Hollow Body Hold	Russian Twist
Hollow Body Hold with Dumbbell	Split-leg Scissors

UBCT exercises were adapted from the works of Mullane et al. [25], Raeder et al. [26], and Balaji and Murugavel [27]. CMST exercises were adapted from the work of Pirmohammadi et al. [16]. UBCT: upper body control training. CMST: core muscle stabilization training.

**Table 2:** General characteristics of the participants.

Variable	UBCT Group (*n* = 21)	CMST Group (*n* = 21)	*p*-Value
Age (year)	24.43 ± 1.46	24.57 ± 1.88	0.786
Gender (M/F)	10/11	9/12	-
Height (cm)	169.09 ± 3.72	168.14 ± 4.94	0.485
Weight (kg)	67.23 ± 3.74	67.04 ± 4.76	0.886
BMI (kg/m^2^)	22.54 ± 1.61	22.77 ± 2.19	0.706
Leg side L/R (num)	5/16	7/14	-
CAIT-K score	19.33 ± 2.51	20.10 ± 1.99	0.284

Data expressed as mean ± standard deviation. UBCT: upper-body-control training, CMST: core muscle stabilization training, M: male, F: female, num: number CAIT-K: Cumberland ankle instability tool–Korean.

**Table 3:** Comparison of muscle activation results within and between groups.

Muscles	Group	Pre-Test	Post-Test	Mean Difference (95% CI)	*t (p)*
GCM	UBCT group	18.57 ± 1.74	20.10 ± 2.80 *	−1.52 (−2.9, −0.0)	−0.299
(%MVC)	CMST group	18.81 ± 1.88	20.05 ± 2.63	−1.23 (−2.6, 0.1)	(0.766)
TA	UBCT group	26.76 ± 0.76	29.05 ± 2.83 *	−2.28 (−3.5, −0.9)	2.488
(%MVC)	CMST group	26.14 ± 1.27	26.71 ± 1.45	−0.57 (−1.1, 0.0)	(0.017 *)
PL	UBCT group	23.38 ± 2.08	24.33 ± 2.43 *	−0.95 (−1.6, −0.2)	0.862
(%MVC)	CMST group	22.29 ± 1.97	22.86 ± 1.90	−0.57 (−1.2, 0.0)	(0.394)

Data expressed as mean ± standard deviation, * *p* < 0.05. TA: tibialis anterior, PL: peroneus longus, GCM: gastrocnemius, UBCT: upper-body-control training, CMST: core muscle stabilization training, MVC: maximum voluntary contraction.

**Table 4:** Comparison of joint movement results within and between groups.

Variables	Group	Pre-Test	Post-Test	Mean Difference (95% CI)	*t (p)*
Ankle EV/IV (°)	UBCT group	1.64 ± 0.77	3.16 ± 0.28 **	−1.51 (−1.8, −1.1)	−1.532 (0.133)
	CMST group	1.78 ± 0.17	2.99 ± 0.50 **	−1.21 (−1.4, −0.9)	
Ankle DF/PF(°)	UBCT group	12.91 ± 0.09	13.11 ± 0.1 1**	−0.20 (−0.2, −0.1)	−0.364 (0.717)
	CMST group	12.84 ± 0.26	13.02 ± 0.19 *	−0.18 (−0.3, −0.0)	
Knee flex/ext (°)	UBCT group	63.52 ± 6.51	65.42 ± 6.74 *	−1.90 (−2.9, −0.8)	−2.204 (0.033 *)
	CMST group	63.61 ± 3.96	64.28 ± 4.25 *	−0.66 (−1.2, −0.0)	
Hip flex/ext (°)	UBCT group	32.71 ± 1.38	33.28 ± 1.92	−0.57 (−1.4, 0.2)	0.819 (0.418)
	CMST group	33.09 ± 1.84	34.52 ± 2.60	−1.42 (−3.4, 0.5)	

Data expressed as mean ± standard deviation, * *p* < 0.05, ** *p* < 0.001. UBCT: upper-body-control training, CMST: core muscle stabilization training, EV/IV: eversion/inversion, DF/PF: dorsiflexion/plantar flexion, flex/ext: flexion/extension.

**Table 5:** Comparison of ground reaction force values within and between groups.

Variables	Group	Pre-Test	Post-Test	Mean Difference (95% CI)	*t (p)*
Peak vGRF(N/kg)	UBCT group	44.14 ± 2.86	40.43 ± 1.74 **	3.71 (2.1, 5.2)	2.074 (0.045 *)
	CMST group	42.05 ± 2.37	40.10 ± 1.67 **	1.95 (1.1, 2.7)	
Time to peak vGRF (ms)	UBCT group	43.38 ± 11.94	58.80 ± 9.01 **	−14.42 (−18,−10)	−2.432 (0.020 *)
	CMST group	53.85 ± 9.64	61.52 ± 11.23 *	−7.66 (−11, −4)	
CAIT-K score	UBCT group	19.33 ± 2.51	21.80 ± 3.81 *	−2.47 (−4.0, −0.9)	−2.083 (0.044 *)
	CMST group	20.10 ± 1.99	20.95 ± 2.29 *	−0.85 (−1.3, −0.3)	

Data expressed as mean ± standard deviation, * *p* < 0.05, ** *p* < 0.001. UBCT: upper-body-control training, CMST: core muscle stabilization training, CAIT-K: Cumberland ankle instability tool–Korean.

**Table 6:** Comparison of ground reaction force results within and between groups.

Variables	Group	Pre-Test	Post-Test	Mean Rank	Z (*p*)
Dynamic balance (cm)					
Anterior	UBCT group	63.00 ± 1.58	64.43 ± 1.63 *	(4.00, 10.60)	−1.754 (0.079)
	CMST group	63.33 ± 2.05	64.00 ± 2.05 *	(8.80, 10.43)	
Posterolateral	UBCT group	81.43 ± 1.32	83.43 ± 1.85 *	(4.50, 11.56)	−2.121 (0.034 *)
	CMST group	81.48 ± 1.28	82.43 ± 1.72 *	(8.50, 10.54)	
Posteromedial	UBCT group	81.95 ± 1.32	83.52 ± 1.86 *	(15.00, 9.18)	−2.205 (0.027 *)
	CMST group	81.90 ± 1.13	82.62 ± 1.53 *	(11.50, 9.60)	

Data expressed as mean ± standard deviation, * *p* < 0.05. UBCT: upper-body-control training, CMST: core muscle stabilization training.

## Data Availability

The data used to support the findings of this study are available from the corresponding author upon reasonable request.

## Supplementary Materials

The following supporting information can be downloaded at: https://www.mdpi.com/article/10.3390/healthcare12010070/s1, Supplementary File S1: Core Muscle Stabilization Training and Upper Body Control Training.
