# Supplementary material for: Comparing the Impact of Upper Body Control and Core Muscle Stabilization Training on Landing Biomechanics in Individuals with Functional Ankle Instability: A Randomized Controlled Trial
Source: Healthcare (Basel). 2023 Dec 28;12(1):70. doi: 10.3390/healthcare12010070 (PMC10778743; doi:10.3390/healthcare12010070)
Supplement: Supplementary file 1 [file healthcare-12-00070-s001.zip › healthcare-2772890-supplementary.pdf]

**Core Muscle Stabilization Training**

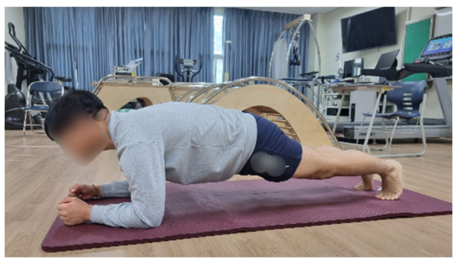

Plank

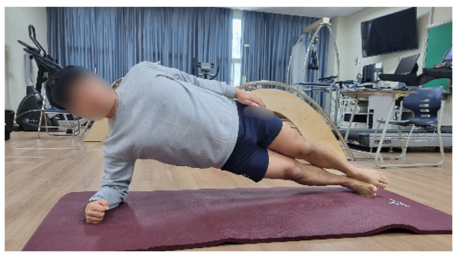

Side plank

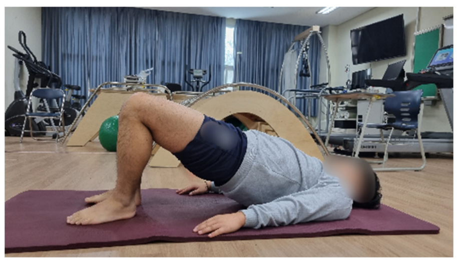

Supine bridge

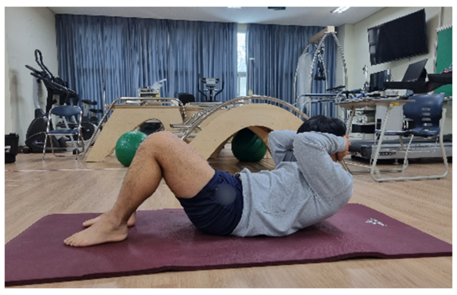

Abdominal crunch

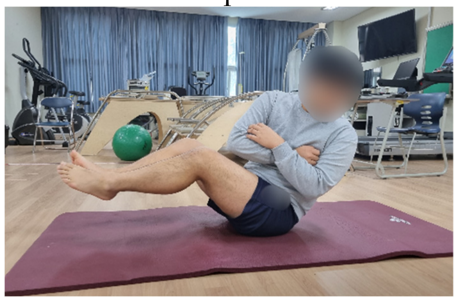

Russian twist

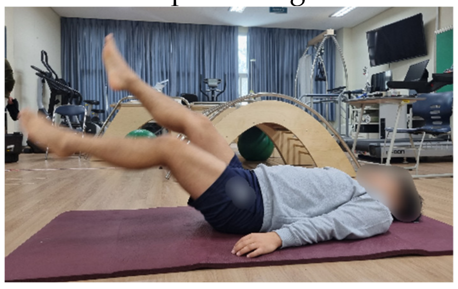

Split leg scissors

**Upper Body Control Training**

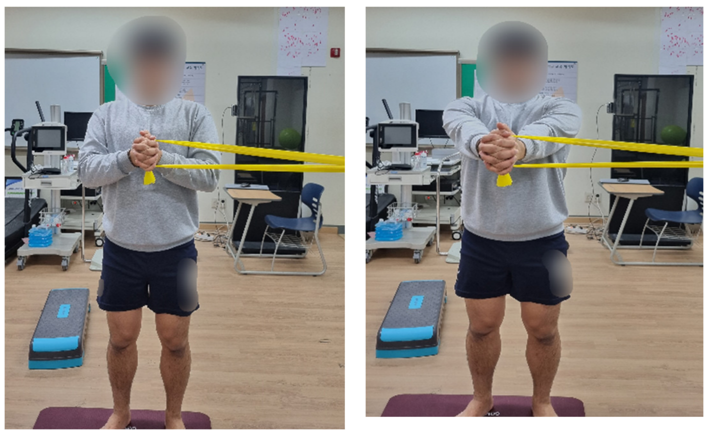

Resistance Band Pallof Press

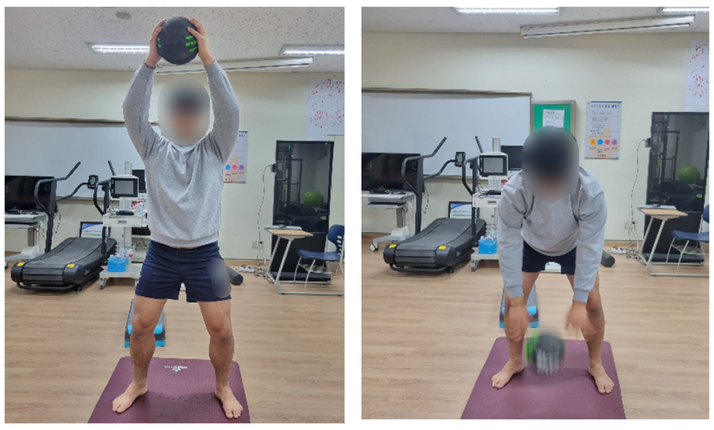

Medicine Ball Slams

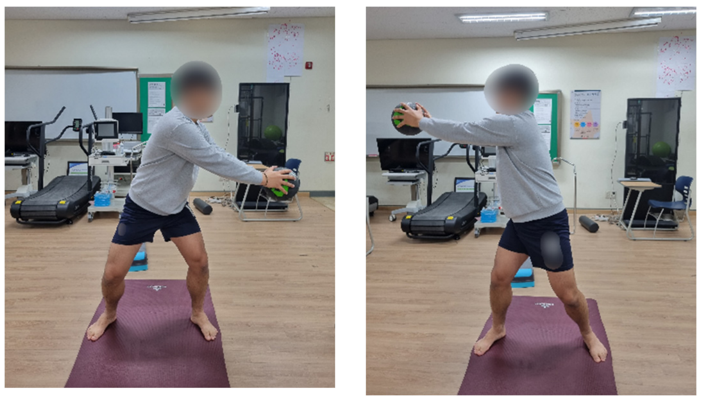

Rotational Medicine Ball Throws

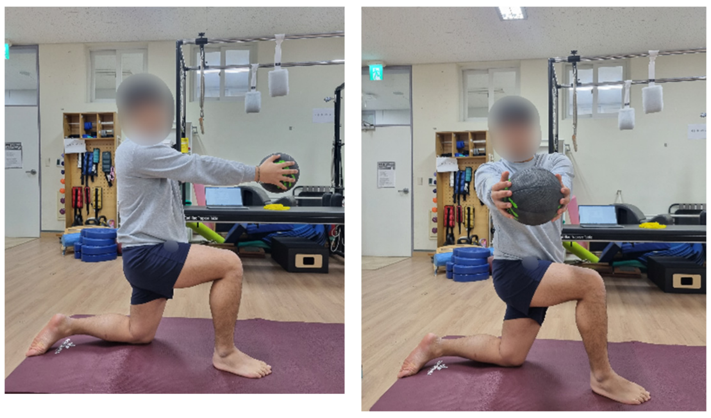

Diagonal Lunge with Twist

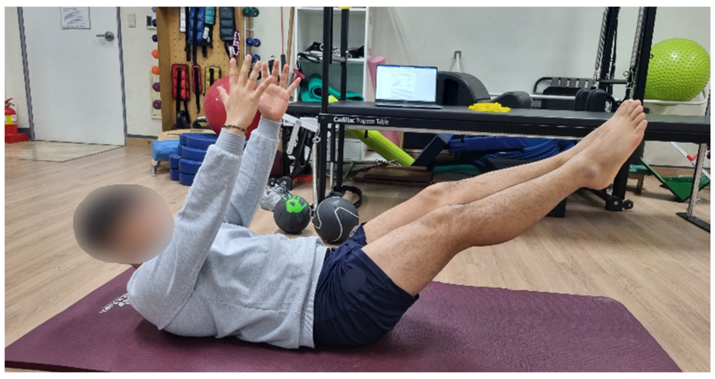

Hollow Body Hold

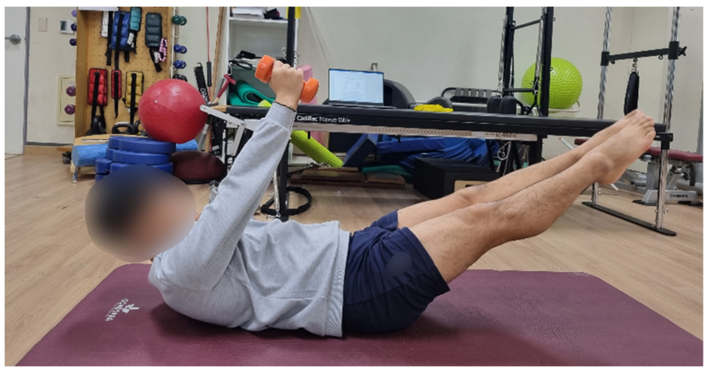

Hollow Body Hold with dumbbell
